# Etiology analysis and computed tomography imaging of a tonsillar inflammatory myofibroblastic tumor: report of an immunocompetent patient and brief review

**DOI:** 10.1186/1758-3284-4-4

**Published:** 2012-03-09

**Authors:** Yun-Zhen Luo, Li-Bo Dai, Shui-Hong Zhou, Xing-Mei Luo, Jun Fan, Ling-Xiang Ruan

**Affiliations:** 1 Department of Otolaryngology, The First Affiliated Hospital, College of Medicine, Zhejiang University, Hangzhou, Zhejiang 310003, China; 2 Department of Otolaryngology, The Second Hospital of Jiaxing City, Jiaxing, Zhejiang Province 314000, China; 3 Key Laboratory of National Infectious Diseases, Institute of Infectious Diseases, First Affiliated Hospital, School of Medicine, Zhejiang University, Hangzhou, 310003, China; 4 Department of Radiology, The First Affiliated Hospital, College of Medicine, Zhejiang University, Hangzhou, Zhejiang 310003, China

**Keywords:** Inflammatory myofibroblastic tumor, Tonsil, Etiology, Computed tomography, Treatment

## Abstract

**Objectives:**

The etiology of Inflammatory myofibroblastic tumor(IMT) is contentious. In this study, we used computed tomography (CT) to examine tonsillar IMT and further analyzed the etiology of this entity.

**Methodology:**

We presented CT features of left tonsillar IMT and reviewed the English-language literature published between 1984 and 2011.

**Results:**

To our knowledge, there are only six published cases of tonsillar IMT including the present case. Two patients were asymptomatic at initial presentation. Two patients were taking immunosuppressants, and one was pregnant and in an immunomodulated state. CT of our patient revealed a 2.6 × 1.8 cm irregular soft tissue mass between the left tonsil and the base of the tongue. It did not invade surrounding structures and was not enhanced on contrast-enhanced imaging.

**Conclusions:**

Tonsillar IMT may be a benign tumor. We suggest that preoperative recognition of tonsillar IMT by CT may be important to avoid unnecessary expanded surgery.

## Background

Inflammatory myofibroblastic tumor (IMT) is a rare mesenchymal tumor[1]. The etiology of IMT is contentious, and debate exists as to whether IMT is benign or malignant[1-3]. Some IMTs are found to be associated with systemic inflammatory disorders[1,4], autoimmune diseases[5], or neoplasms[6]. Its prognosis and behavior are controversial. Most patients with IMT have a good prognosis and can be cured by resection. Recent evidence shows that IMTs may have different etiologies and clinicopathologic features at different sites[3,7,8].

Tonsillar IMT is rare; we reviewed the English-literature regarding tonsillar IMT and only five previous reports covered this topic[9-13]. Two of the five patients were taking immunosuppressants, and one was pregnant and in an immunomodulated state. We herein report the sixth case of tonsillar IMT in the English-language literature in an immunocompetent patient and first describe the computed tomography (CT) imaging features of tonsillar IMT.

## Case presentation

**Figure 1:**
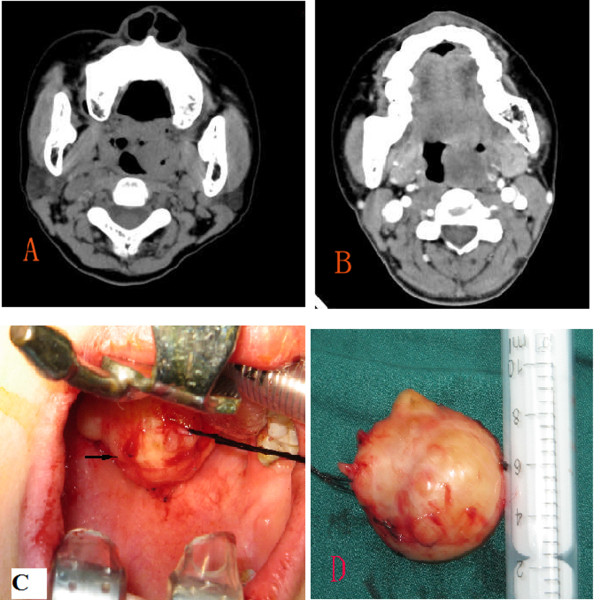
**CT of pharynx showed that a 2.6 cm × 1.8 cm irregular soft tissue mass between the left tonsil and the base of the tongue(A), with no enhanced on contrast-enhanced imaging(B)**.C: The mass and left tonsil were excised completely. D: surgical specimen.

A 51-year-old woman presented with a 3-year history of a progressively enlarging mass in the left palatine tonsil. The mass had been approximately 0.5 × 0.5 cm during a routine check-up 3 years previously. The patient denied sore throat, fever, malaise, dysphagia, and dyspnea. The patient had no history of generalized immune deficiency, leukemia, malignant disease, diabetes mellitus, or use of immunosuppressive drugs, including corticosteroids. The patient also denied alcohol and tobacco abuse and any previous surgery or trauma. The patient had not visited a doctor for evaluation of the mass. Six months prior to presentation, the patient felt a dry itch in her throat and the mass had progressively enlarged. In June 2011, the patient presented to our department. Physical examination showed a 2.5 × 2 cm irregular mass in the upper pole of the left palatine tonsil. There was no adenopathy. The rest of the physical examination results were normal. CT of the pharynx showed a 2.6 × 1.8 cm irregular soft tissue mass between the left tonsil and the base of the tongue (Figure 1A) with no enhancement on contrast-enhanced imaging, and no enlargement of the lymph nodes (Figure 1B). Laboratory examination revealed a normal complete blood count and normal erythrocyte sedimentation rate. Serological tests were positive for anti-Epstein-Barr virus (EBV) IgG and anti- cytomegalovirus (CMV) IgG, and negative for anti-EBV IgM, anti-CMV IgM, and anti-human immunodeficiency virus (HIV) antibodies. There were no other abnormalities on systemic or dermatological examinations. Chest X-ray and abdominal ultrasonography (USG) results were normal.

**Figure 2:**
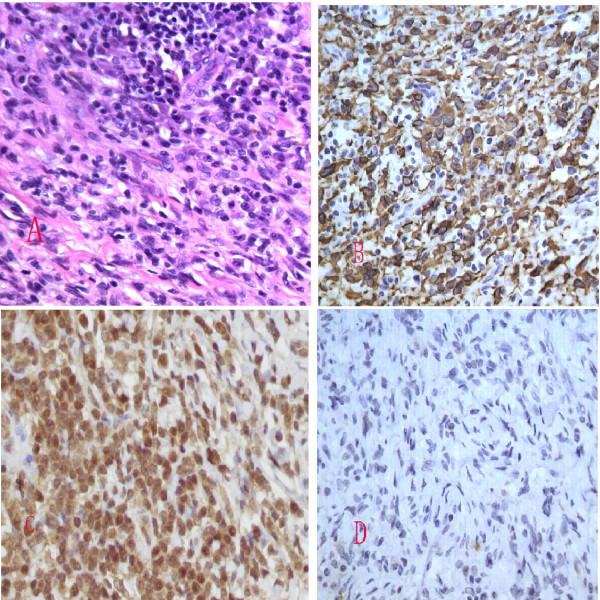
**Routine pathological examination demonstrated that the tumour was composed of short spindle cells arranged as weaving**. The spindle cells in some areas were rich and some cells were atypia and mitotic, mixed with inflammatory cells such as lymphocytes and plasma cells arranged as cluster(A:HE × 40). Immunohistochemical analysis was positive for the expression of desmin(B: EliVision × 40) and AKT(3 C: EliVision × 40), focal positive for S-100 protein(D: EliVision × 40).

On June 17, 2011, the mass and left tonsil were excised completely under general anesthesia without any complications (Figure 1C). Pathological analysis of frozen sections showed an inflammatory pseudotumor. Routine pathological examination demonstrated that the tumor was composed of short spindle cells arranged in a weaving pattern. The spindle cells in some areas were rich; some cells showed atypia and mitosis and were mixed with inflammatory cells, such as lymphocytes, and plasma cells arranged as clusters (Figure 2A). Immunohistochemical (IHC) analysis was positive for the expression of desmin (Figure 2B), and focally positive for S-100 protein. In contrast, IHC analysis was negative for CD117, CD34, smooth muscle actin, caldesmon, and cytokeratin. Further molecular pathological investigation by polymerase chain reaction (PCR) did not reveal CMV or EBV infection. There was no evidence of recurrence after 8 months of follow-up.

## Conclusions

IMT may occur at nearly every site of the body[8], including the lungs[3], hypopharynx [4], central nervous system[5], abdomen[7], orbit[7], extremities[7], and oral cavity[8]. Tonsillar IMT is rare. In 1984, Weilbaecher et al. were the first to report IMTs of the tonsil[12]. We reviewed the English-language literature regarding IMTs of the tonsil from 1984 to 2011, and only six cases were reported (including the case described herein) (Table 1)[9-13]. The patient group consisted of two males and four females. The age of the patients ranged from 10 to 63 years at initial presentation, with a mean age of 43 years. The symptoms included sore throat, odynophagia, pain in the neck region, cough with vomiting, and dyspnea (in a young female). Two patients were asymptomatic at initial presentation, one had only an enlarged tonsil, and one had a mass in the left tonsil. There were four cases with IMT located in the left tonsil and two cases with IMT in the right tonsil.

**Table 1:** Clinical Features of IMT Cases in the tonsil

Author	Age/sex	past history	Symptom	Size (cm)	Duration	Treatment	Follow-up
Present	51/F	no past medical history of note	A progressive enlarged mass in the left tonsil	2.6 × 1.8	3 yrs	Tonsillectomy (left)	3 m, NED
Grube-Pagola et al (2011)^12^	10/F	no past medical history of note	pain in the neck region, cough with vomiting, dyspnoea and dyslexia	5.2 × 2.4 × 1.5	11 m	Bilateral tonsillectomy (lesion in the left tosil)	NA
Magill et al (2010)^13^	28/F	pregnant woman	sore throat, fever and malaise	NA	10-day	CO_2 _laser (right)	13 m, NED
Gangopadhyay et al (1997)^14^	41/M	chronic renal failure received a cadaveric renal transplant, HCV+, prednisone, azathioprine, enalapril cyclosporine since renal transplant.	lump in the throat, odynophagia	2 × 2 × 2	2 m	Tonsillectomy (left)	10 m, NED
Newman et al (1995)^15^	62/F	Asthma, retroperitoneal fibrosis, Prednisone(o.d)	odynphagia	3.5 × 2.5 × 2	2 m	Tonsillectomy (left)	16 m, NED
Weilbaecher et al (1984)^16^	63/M	no past medical history of note	an enlarged right tonsil	2.5 × 1.5 × 1	NA	Tonsillectomy (right)	NA

*NA *not available; *NED *no evidence of disease.

Although debate exists as to whether IMT is a pseudotumor or a neoplasm, the concept of IMT as a neoplasm was recently solidified [7] with the discovery of cytogenetic aberrations and the subsequent recognition of ALK gene rearrangements on the short arm of the chromosome as a recurrent aberration[8]. However, the etiology, benign or malignant nature, and prognosis of IMT remain unknown and controversial. Our previous reports and other studies revealed that IMTs originating in different organs may lead to diverse etiologies and clinicopathologic features[2,3,7,8]. Etiologies at the other sites may be infectious[14], traumatic[15], or autoimmune syndromic[5]. Viruses that are suspected to be involved in the development of IMTs include HIV[16], HHV-8[4], and EBV[17]. Some investigators consider it to be an immunologic response to an infectious agent[16,17]. For tonsillar IMT, two patients were in an immunodepressive state[11,12] and one was a pregnant woman[10]. The patient with IMT of the tonsil, reported by Newman and Shinn (1995), had been on long-term prednisone for asthma and retroperitoneal fibrosis[12]. Gangopadhyay et al. reported that the patient had received long-term prednisone and other immunosuppressants following cadaveric renal transplant 9 years previously[11]. Magill et al. reported a pregnant woman with IMT in the right tonsil[10]. They suggested that pregnancy is an immunomodulated state, even during the earliest gestational period[10]. The present patient was immunocompetent.

Magill et al. mentioned that CT confirmed the presence of an enlarged right tonsil along with right-sided nodes, but did not supply any CT imaging[10]. Here, we first describe CT imaging of tonsillar IMT. CT revealed a 2.6 × 1.8 cm irregular soft tissue mass between the left tonsil and the base of the tongue that did not invade surrounding structures and was not enhanced on contrast-enhanced imaging. These findings suggested that the mass might have been a benign tumor. The results were confirmed by surgery and pathology. The surgical specimen showed a smooth surface with some small nodules, which lead to the appearance of an irregular soft tissue mass on CT imaging. Thus, preoperative recognition of this benign lesion on CT is important to avoid unnecessary radical resection. Our imaging findings were not similar to those of other IMTs in the head and neck[18]. Most IMTs on contrast-enhanced images show early enhancement, and sinonasal IMTs have a more aggressive appearance[18]. On CT, a moderately enhanced mass is usually seen in orbital IMTs, accompanied by fat infiltration or edema[18]. Our case showed no enhancement on contrast-enhanced imaging and did not involve surrounding tissue.

IMTs arising in mesenteric, omental, peritoneal, pelvic, and retroperitoneal sites[19], and the maxillary sinus[20] tend to recur, with a potential for metastatic spread in rare instances[2,3,20]. In the six tonsillar IMTs reported, there were no recurrences or metastases. This suggests that tonsillar IMTs may be benign and have a good outcome. However, the prognosis, recurrence, and metastatic rate have not been confirmed in a larger series, and there is a lack of long-term follow-up.

The optimal treatment for IMTs at other sites is still controversial, but excision of the tonsils with IMT, either by tonsillectomy or excision with a CO_2 _laser, seems to be an effective treatment based on the outcome of four tonsillar IMTs[10-12]. Treatment experience will continue to accumulate along with increases in the number of tonsillar IMTs.

## Consent

Written informed consent was obtained from the patient for publication of this Case report and any accompanying images. A copy of the written consent is available for review by the Editor-in-Chief of this journal.

## Abbreviations

IMT: Inflammatory myofibroblastic tumor; CT: Computed tomography; EBV: Epstein-Barr virus; HIV: Human immunodeficiency virus; USG: Ultrasonography; IHC: Immunohistochemical; PCR: Polymerase chain reaction.

The English in this document has been checked by at least two professional editors, both native speakers of English. For a certificate, please see: http://www.textcheck.com/certificate/nSTlES

## Competing interests

The authors declare that they have no competing interests.

## Authors' contributions

Yun-Zhen Luo designed the manuscript. Li-Bo Dai and Xing-Mei Luo aided in the surgery and collected the materials. Shui-Hong Zhou performed the surgery and wrote the manuscript. J un Fan did the laboratory examination. Ling-Xiang Ruan analysed the imaging of CT. All authors read and approved the final manuscript.
